# A happy home? Socio-economic inequalities in depressive symptoms and the role of housing quality in nine European countries

**DOI:** 10.1186/s12889-023-17070-z

**Published:** 2023-11-08

**Authors:** Brendan McElroy, Edel Walsh

**Affiliations:** Dept of Economics, Cork University Business School, University College Cork, Cork, Ireland

**Keywords:** Health inequalities, Depression, Concentration index, Housing quality, SDG3 good health and well-being, SDG10 reduced inequalities.

## Abstract

**Background:**

This study examines the prevalence of and socio-economic inequalities in depressive symptoms in nine high-income European countries, focusing in particular on the role of housing quality.

**Methods:**

Using the European Social Survey, a concentration index of depressive symptoms in each country is estimated. The role of housing quality is assessed by examining the risk factors associated with the concentration index, using the Recentred Influence Function method. To contextualise the housing quality results, other predictors of inequalities in depressive symptoms inequalities are also quantified and discussed.

**Results:**

Our results indicate that inequalities in depressive symptoms are concentrated among poorer respondents both in each country and in total. Austria and Belgium have the lowest inequalities and France has the highest. No geographic pattern is evident. Housing problems are associated with higher inequalities in six of the nine countries in the sample. While no association is evident for indicators of socio-economic status such as years of education and income, financial strain is significant.

**Conclusions:**

This study is the first to estimate the degree of socio-economic inequality in depressive symptoms across European countries. The association between poor housing and poorer inequalities suggests that housing has a role to play lowering depressive symptoms inequalities.

**Supplementary Information:**

The online version contains supplementary material available at 10.1186/s12889-023-17070-z.

## Background

This study has two aims. The first is to compare socio-economic inequalities in depression across high income countries in Europe. The second is to assess the relationship between housing quality and inequalities in depression. The study uses the European Social Survey (ESS) to estimate the prevalence of depressive symptoms and a concentration index of inequality in depressive symptoms in each of nine European countries. Risk factors for depressive symptoms and inequalities in depressive symptoms are quantified, focusing particularly on the contribution of housing quality. To put the housing quality results into context, and to provide a better understanding of the role of all risk factors relating to inequalities in depressive symptoms, we also examine the other variables in the models.

Persistent differences in health by socio-economic status provide key policy challenges facing many countries in Europe [1], and calls have been made for purposeful strategies aimed at reducing inequalities in mental health conditions such as depression [2]. It is estimated that 7.2% of people in the EU suffer from chronic depression [3] leading to significant economic, social and individual costs. Income inequalities in mental health mean that relatively poorer individuals are more likely to experience mental health conditions, such as depression, than those not defined as poor [4]. The extent to which socio-economic determinants explain inequalities in depression is a concern for health policy makers in establishing informed policies to reduce them [5]. Moreover, there is a need to better understand the individual, social and economic risk factors that contribute to depression inequalities and the possible role that housing quality may play in shaping depression inequalities.

According to the World Health Organisation, housing conditions can be considered as one of the mechanisms through which social inequality translates into health inequalities [6], and while social inequalities in health, in the EU and elsewhere, have been widely studied, empirical studies examining the effect of poor housing on socio-economic health inequalities are uncommon [7]. Exceptions include Nie et al. (2022) and Urbanos-Garrido (2012) who find evidence, in single-country studies, that housing deprivation is positively associated with income-related poor-health inequality [8, 9]. Nie et al. (2022) examine the role of housing in income-related health inequalities in urban China and find that better housing conditions reduce income-related inequalities in self-assessed health and objective measures of physical health (obesity, high blood pressure) [8]. Using cross-sectional Spanish data, Urbanos-Garrido (2012) examine the impact of housing deprivation on health inequalities using three binary health measures; self-assessed health, the presence of a chronic condition or the presence of a health problem that limits daily activities [9]. There is evidence to suggest pro-rich inequality in health, with housing accounting for between 7.17% and 13.85% of income related health inequality [9]. Chan et al. (2022) find evidence of mental health inequalities in Hong Kong especially among those living in public rental housing that may be of poorer quality [10].

Attempts to establish a causal pathway from poor housing to ill-health are evident in the current literature. For example, Pevalin et al. (2008) use seven waves (1996–2002) of the British Household Panel Survey (BHPS) and find that an increase in housing problems increases the number of general health problems for both men and women, and worsens mental health for older men [11]. Navarro et al. (2010) use a housing deprivation index to measure dwelling problems and estimate the relationship with poor self-assessed health using four waves (1995–1998) of European Community Household Panel (ECHP) data for Spain [12]. After controlling for both observed and unobserved heterogeneity among individuals, the results suggest that moving up to the next score of the housing deprivation scale increases the probability of self-reported bad health by 80%. Using European panel data from the EU-SILC, Angel and Bittschi (2019) estimate fixed effects models and provide evidence that deprived living conditions have a direct causal effect on health [13]. Evidence links specific housing issues such as an inability to keep the dwelling warm and structural dwelling problems, with significant adverse health effects [12, 13]. Using longitudinal data (1985–2009) on British civil servants, Howden-Chapman et al. (2011) report housing quality to be a significant factor in explaining the mental health (GHQ scores) of older people, and the impact of housing problems becomes more important (than home ownership) as people get older [14].

In terms of mental health, income is found to account for more than 50% of inequality in reported depression [15]. However, other factors such as labour status, education and demographics have been found to contribute to socio-economic inequalities in depression [5, 16, 17]. Linder et al. (2020) document an increase in the income related inequality in psychiatric diagnoses in Sweden from 1994 to 2011 and find that these are increasingly concentrated among the lower educated and lower income groups [18]. In China, Xu et al. (2016) decomposed the concentration index of depressive symptoms in the elderly, finding that these were concentrated among poorer individuals [19].

The relationship between housing and mental health inequalities is complex. Experiences of poor housing conditions are not evenly distributed across populations and those on lower incomes, for example, tend to be disproportionately affected [20]. Exposure to poor housing conditions (damp, mould, cold) may also exacerbate existing mental health inequalities [21]. Targeted measures aimed at improving poor housing conditions are often recommended to tackle health inequalities [22]. Several systematic reviews of housing interventions report evidence of mental health improvements following home improvements [23–26]. Housing mobility policies, where disadvantaged residents are moved to an area experiencing less poverty, can lead to a decrease in the numbers reporting depression and an increase in those reporting good or excellent health [27, 28]. Hence, through longitudinal, panel and specific intervention evaluations there is a good degree of literature to support a causal pathway from housing conditions to health.

This study contributes to several strands of literature, including estimating inequalities in depression, analysing the role of housing quality in depression inequalities and providing evidence on the contribution of other risk factors to depression inequalities.

## Methods

### Factors associated with depressive symptoms

The depression measure used in this study is a count of depressive symptoms, which we model using a Negative Binomial distribution, similar to Chen et al., (2023) [29] as follows:

1 $${y}_{ij}\sim NB({\mu }_{ij}, \alpha )$$

where ${y}_{ij}$ is individual *i*’s from country *j*’s count of depressive symptoms, ${\mu }_{ii}$ is the mean of ${y}_{ij}$ conditional on the vector of covariates and $\alpha$ is an overdispersion parameter measuring the extent to which variance exceeds the mean. The conditional mean term ${\mu }_{ij}$ is modelled as:

2 $${\mu }_{ij}=exp(\alpha {HQ}_{ij}+\beta {C}_{j}+\gamma {HQ}_{ij}{.C}_{j}+{\delta Z}_{ij})$$

where *HQ*_*ij*_ is housing quality, *C*_*j*_ is country of residence, *HQ*_*ij*_.*C*_*j*_ is the interaction effect between housing quality and country of residence, *Z*_*ij*_ is the set of demographic, socio-economic, early-life and community covariates, $\alpha ,\beta ,\gamma ,\delta$ are vectors of coefficients to be estimated. For ease of interpretation, the marginal effect of the combined main housing quality effect and the interaction effect, calculated using Stata’s *margins* module [30], is presented.

### Socio-economic inequality in depressive symptoms

This study measures socio-economic inequality in depressive symptoms using the concentration index (CI) [31]. The concentration curve plots the cumulative proportion of the population ranked by some measure of socio-economic status (in this, as with most cases, income) against the cumulative proportion of ill-health (in this case depressive symptoms). The concentration index calculates the degree of inequality as twice the area between the concentration curve and the line of equality, which can be expressed as twice the covariance of depression and ranked income as follows (where subscripts *i* and *j* are suppressed):

3 $$CI=2cov(y,R)$$

where *R* is rank on a cumulative scale in income ranging from 0 to 1.

The concentration index requires that the health variable is on the same scale as the ranking variable, and since income is ratio scale with no upper bound and our health variable has a theoretical upper bound of 24, our concentration index needs to be corrected. Erreygers (2009) proposed a weighting to the concentration index that allows bounded health variables to be employed [32]. The Erreygers index (EI) employs the following correction:

4 $$EI= \frac{4\mu }{{h}_{max}-{h}_{min}}CI$$

Where *h*_*max*_, *h*_*min*_ are the upper and lower bounds of the health variable.

One of the strengths of using the same dataset in multiple countries is that comparisons are not contaminated by differences in data capture in different countries. The comparison of both point estimates and tests for statistically significant differences is thereby facilitated. This supports the study’s first aim.

### Factors associated with Socio-economic inequalities in depressive symptoms

Decomposing the concentration index following the method proposed by Wagstaff et al., (2003) [33] is commonplace [9, 34–36], but it has been subject to a number of criticisms. These focus primarily on the fact that it explains the variance in health rather than the covariance between health and socio-economic rank, as per Eq. (3), and on the number of restrictive assumptions that it imposes (see Cai et al., (2017) and Heckley et al. (2016) for more comprehensive reviews [37, 38]). In response, Heckley et al. (2016) implemented a recentred influence function (RIF) regression for health inequality [38]. The RIF has several advantages over the Wagstaff et al. (2003) decomposition [33]. The RIF is a transformation of the concentration index that generates a RIF value for each respondent. It accounts for the influence of the removal of an observation on the estimated index. The average of the RIF is the concentration index meaning that the factors associated with inequality can be explicitly modelled. We use the same set of covariates as in (2), as follows:

5 $${RIF}_{ij}=\alpha {HQ}_{ij}+\beta {C}_{j}+\gamma {HQ}_{ij}{.C}_{j}+{\delta Z}_{ij}+{\epsilon }_{ij}$$

Where RIF is the respondent’s RIF value. Coefficients are interpreted as marginal effects with a negative (positive) coefficient worsening (improving) inequalities [38].

RIF regression has been applied by Heckley et al. (2016), Cai et al. (2017), Linder et al. (2020) and Nie et al. (2022) [8, 18, 37, 38]. We estimate RIF regressions using Stata’s *rifhdreg* module [39].

## Data

Data from the European Social Survey (ESS) are used in this study. The ESS is a large-scale, cross-sectional survey administered across Europe and Israel, every two years since 2002. It includes two main sections; a core module which is consistent from round to round, and a second section containing two or more rotating modules which are repeated at intervals. The core module contains a comprehensive set of personal and socio-economic variables. We use ESS Round 7 (ESS7) from 2014/15 as it contains a rotating module on social inequalities in health, including questions on housing quality and on depressive symptoms [40]. Post-stratification probability weights are used for all analysis, which was conducted in Stata 16 [30].

### Choice of country

Whilst income is our measure of socio-economic status, income and its distribution vary considerably in Europe and in the ESS. Income level is an important exclusion criterion because the income elasticity of depression may be different at different levels of income. Hence, we choose a group of countries with a median equivalent income of between €20,000 and €30,000. Income distribution matters because income inequality is related to health inequality [1]. We choose Gini coefficients between 0.20 and 0.30. The countries of choice are presented in Table 1. We included Denmark as its median income was just above the threshold and Great Britain as its Gini was just above the threshold. Countries analysed include 3 Nordic countries and 6 North European countries. Supplemental File 1 provides further justification for the choice of these levels of income and income inequality.

**Table 1:** Countries with similar median income and Gini coefficient

Country	Median (€)	Gini
Austria	22,476	0.243
Belgium	20,436	0.247
Germany	22,703	0.269
Denmark	31,583	0.257
Finland	26,493	0.252
France	20,380	0.277
Great Britain	25,153	0.318
Netherlands	23,200	0.247
Sweden	29,039	0.233

Source: ESS, 2015

### Variable description

Depression is measured by the Centre for Epidemiologic Studies Depression Scale – CES-D 8 [41]. We calculate a composite score by summing the eight item responses and implementing a 0–24 point scale, increasing in depressive symptoms. Respondents are asked questions related to feelings of depression, effort, happiness, restless sleep, loneliness, enjoying life, feeling sad and inability to get going, in the week preceding the questionnaire. Its validity has been demonstrated in Missine et al. (2014) and Schane et al. (2008) [42, 43] and has been applied in such recent literature as Cao et al. (2022) and Bracke et al. (2020) [44, 45]. A binary variable version of the CES-D 8 scale has also been used as a marker of depression, where above a chosen threshold score respondent’s are indicated as having depression, and below that threshold they are indicated as having no depression. The threshold has varied in the literature from 7 to 10. Because it uses the full information set and does not depend on a somewhat arbitrary threshold, we use the full CES-D 8 score.

Our principal covariate of interest, poor housing quality, measures any problems with the respondent’s accommodation, varying from rot, mould, dampness, leaking roof, extremely hot or cold, lack of basic plumbing or overcrowding. It is a binary variable with 1 indicating the presence of any of these problems [7, 46–48].

Several control variables are included in the analyses because previous research indicates an association with depression. For example, age and age squared, gender (male = 1), number in the household, presence of children in the household (= 1), and marital status (= 1 if married or in a civil partnership) are included [44, 49]. We further include a number of socio-economic determinants of mental health disorders namely, years of full-time education completed, equivalised income, financial strain and main activity [4, 50]. In some covariates, categories are merged owing to low frequencies or to better summarise their effect.

The ESS measures annual income at household level in country-specific income deciles. These were converted into Euro values. Exchange rates for countries outside the Eurozone were provided in ESS supporting documentation. For those countries that reported an open-ended top decile, we used the fixed multiple approach [51–53], whereby the average income in the top decile was the value at the top of the 9th decile multiplied by 1.3. This is a conservative estimate of the top of the income distribution. The absolute values of household income deciles at country level are adjusted for variations in household composition by applying the square root equivalence scale [54]. For the regression functions reported below, equivalent income is entered in natural logarithmic form.

Financial strain is a categorical variable varying in severity from ‘living comfortably on current income’ to finding it ‘very difficult on current income’. The two most severe categories are merged owing to their low frequencies. Main activity includes paid employment, education, ‘welfare’, which merged two original categories (unemployment and disabled), retired and ‘other’. ‘Other’ includes military service, housework and ‘other’ and were merged due to their low frequencies.

Exposure to stressors in early life can lead to poorer mental health outcomes and evidence suggests that childhood adversity is a key risk factor for the onset and persistence of mental health disorders [55]. In this study, early-life experience variables include severe financial difficulties in the family when growing up, and serious conflict in the household when growing up. As the questions related to potentially traumatic events in both instances, a binary variable was created with ‘always’, ‘often’ and ‘sometimes’ categories indicating presence and ‘hardly ever’ and ‘never’ indicating absence (as per Pérez-Hernández et al., 2019 [48]). Preliminary analysis included paternal and maternal educational attainment. The response rate to these two questions was very low, however, and their effects were statistically insignificant. Since listwise deletion was employed in regressions, we chose to exclude them from the analysis to maximise the sample size available.

Following existing literature, we also include several control variables for the community/area of residence and individual social capital owing to their associations with depression [56–61]. These variables include number of close friends, low engagement in social activities, generalised trust, being victim of a burglary or assault, and perception of safety of local area for walking after dark. In this study, number of close friends is a constructed metric variable from the seven category variable ‘number of people with whom you can discuss personal or intimate matters’. The first four categories were assigned values equal to their initial values; category ‘4–6’ was assigned a value of 5; and ‘7–10’ was assigned 8.5. The highest category, ‘10+’, was opened-ended. It contained a substantial 5% of the sample and so we assumed that there was a high number of additional values here and assigned a value of 13. Low engagement in social activities is a dichotomous variable, constructed from the variable ‘participation in social activities compared to others of same age’. Here we assigned a value of 1 to below average (‘much less’ and ‘less’) and 0 to average and above (‘about the same’, ‘more than most’, ‘much more than most’).

Research has found that individuals with lower generalized trust and weaker social trust in their neighbourhood have a higher probability of suffering from depressive symptoms [62]. Not surprisingly, being the victim of a crime has a significant impact on increasing the risk of developing or exacerbating depressive symptoms [63, 64] and particularly long-term depression [65]. In this study we employ a widely used measure of generalised trust based on a score response scale of 0 to 10; where 0 means ‘you can’t be too careful’ and 10 means that ‘most people can be trusted’. Victim of burglary/assault is a dichotomous variable where the respondent is asked if they or a household member was a victim of burglary or assault in the previous 5 years. ‘Area safe’ is a dichotomous variable based on the 4 category variable ‘Perception of safety of local area for walking after dark’. We assigned ‘1’ to ‘very safe’ and ‘safe’ and ‘0’ to ‘unsafe’ and ‘very unsafe’.

## Results

Results are presented in three parts. The first part reports the findings on the inter-country differences in the prevalence of depressive symptoms and inequality in depressive symptoms. The second part describes the relationship between housing quality and depressive symptoms / inequality in depressive symptoms. The third part provides context to the results of part 2, by reporting on the other covariates in Eqs. (2) and (5).

### Country-level prevalence of depressive symptoms and inequalities in depressive symptoms

**Fig. 1:**
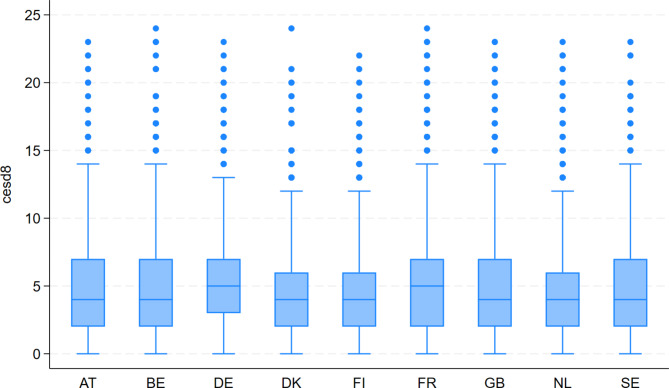
Box plot of depressive symptoms (CES-D 8) scores by country. Source: ESS, 2015. Authors’ calculations. AT = Austria; BE = Belgium; DE = Germany; DK = Denmark; FI = Finland; FR = France; GB = Great Britain; NL = Netherlands; SE = Sweden

Figure 1 reports summary statistics for CES-D 8 by country. As CES-D 8 is a positively skewed count variable, the median and related distribution statistics are more appropriate than the mean and related statistics to summarise the data across countries. The median is four for all but France and Germany, both of which are five. Interquartile ranges are largest in Austria, Belgium, France, Great Britain, Sweden; smallest in Germany, Denmark, Finland and the Netherlands.

**Fig. 2:**
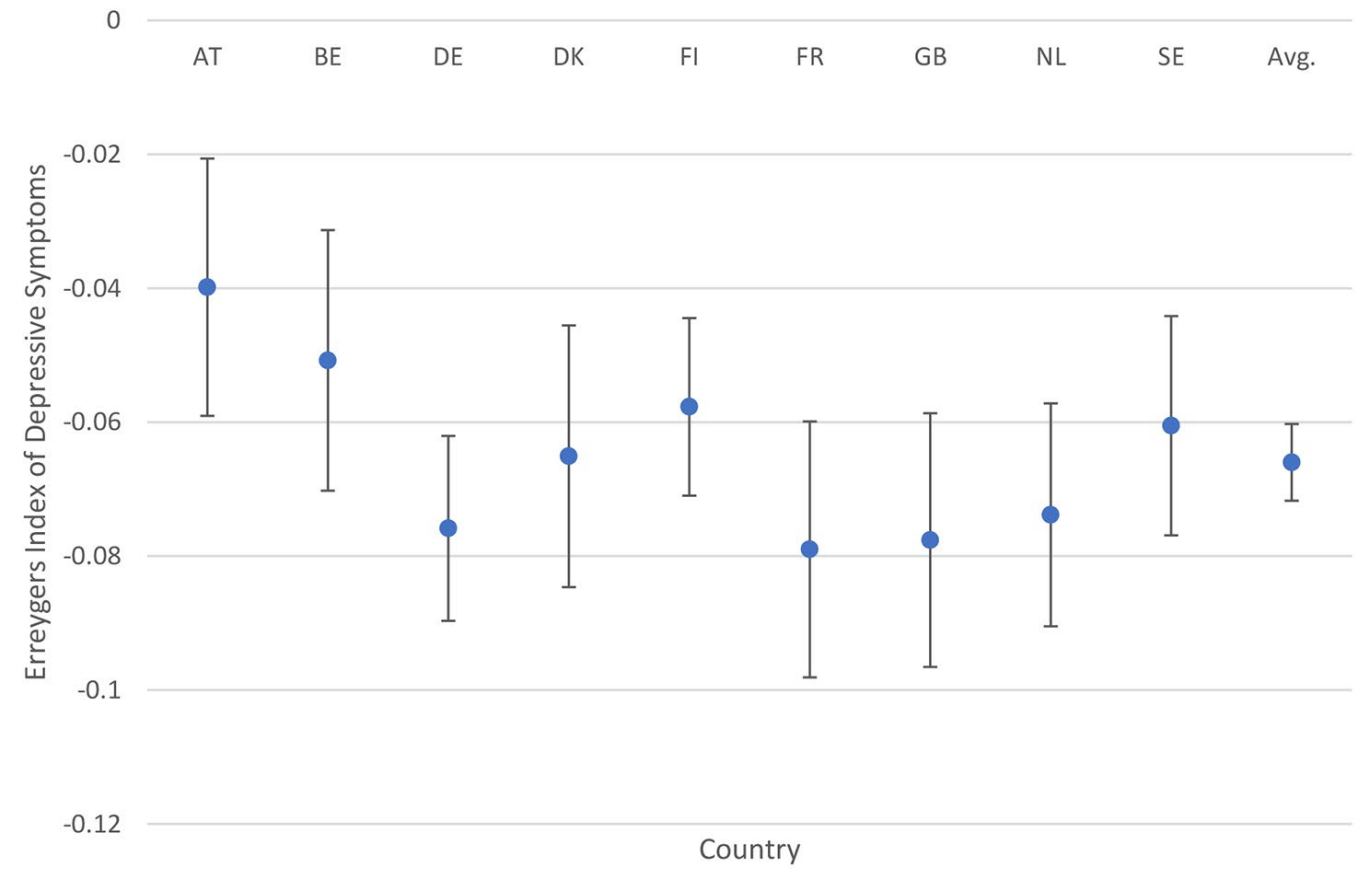
Erregygers index of depressive symptoms. Source: ESS, 2015; Authors’ calculations. AT = Austria; BE-Belgium; DE = Germany; DK = Denmark; FI = Finland; FR = France; GB = Great Britain; NL = The Netherlands; SE = Sweden

Table 2 reports the Erreygers Index by country and the pooled average while Fig. 2 presents these statistics graphically.

**Table 2:** Erreygers index of depressive symptoms (CES-D 8) by country

Country	EI	95% CI	
Austria	-0.03985	-0.05907	-0.02064
Belgium	-0.05078	-0.07024	-0.03133
Germany	-0.07587	-0.08968	-0.06207
Denmark	-0.06509	-0.08463	-0.04555
Finland	-0.05771	-0.07096	-0.04445
France	-0.079	-0.09811	-0.05989
Great Britain	-0.07761	-0.09655	-0.05867
Netherlands	-0.07384	-0.09049	-0.05719
Sweden	-0.06053	-0.0769	-0.04415
Pooled Average	-0.0660	-0.07173	− 0.060265

Source: ESS, 2015; Authors’ calculations; EI = Erreygers Index

The pooled-average EI is -0.0660, meaning that there is a pro-poor concentration of depressive symptoms across all countries. It varies from least pro-poor in Austria (-0.0399), followed by Belgium (-0.0508), and most pro-poor in France (-0.0790) and Great Britain (-0.0776). Tests of equality of means indicate that all countries are statistically equal at the 5% level of significance, except for Austria and Germany which are not statistically equal (P > F = 0.00).

### Housing quality and depressive symptoms / inequalities in depressive symptoms

**Fig. 3:**
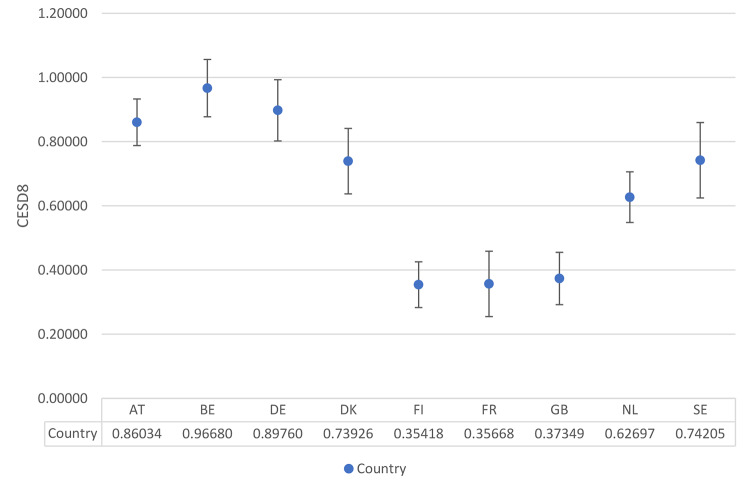
Marginal association between housing quality and depressive symptoms. Source: ESS, 2015; Authors’ calculations. AT = Austria; BE-Belgium; DE = Germany; DK = Denmark; FI = Finland; FR = France; GB = Great Britain; NL = The Netherlands; SE = Sweden

The average marginal association between housing quality and depressive symptoms varies by country, as exhibited in Fig. 3. Three groups emerge, - there is the highest association between the two in Austria, Belgium and Germany and the lowest association in Finland, France and Great Britain. Mid-ranking associations occur in Denmark, the Netherlands and Sweden. Overall, living in a poor-quality house is associated with an increase in CES-D 8 of between approximately a third of a unit and one unit.

**Fig. 4:**
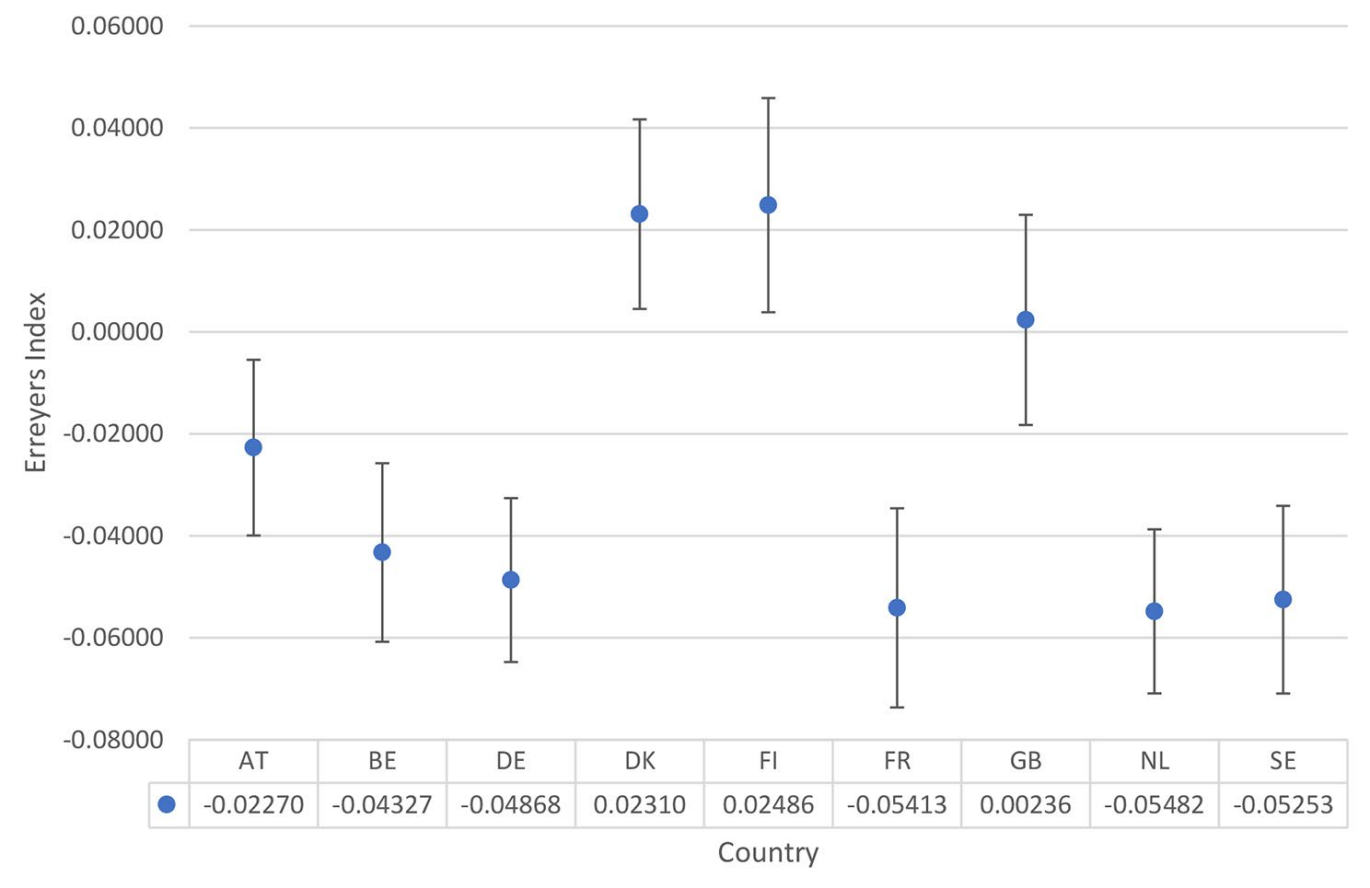
Marginal association between housing quality and inequality in depressive symptoms. Source: ESS, 2015; Authors’ calculations. AT = Austria; BE-Belgium; DE = Germany; DK = Denmark; FI = Finland; FR = France; GB = Great Britain; NL = The Netherlands; SE = Sweden

The marginal association of poor housing quality and depressive symptoms inequality is negative in six of the nine countries studied (meaning greater inequality) of between approximately − 0.022 and approximately − 0.054 (Fig. 4). These countries are, in rank order of (negative) point estimates, the Netherlands, France, Sweden, Germany, Belgium and Austria. A visual inspection of Fig. 4 indicates, however, that all six are insignificantly different from each other. Poor housing quality is associated with lower inequalities in Denmark and Finland while in Great Britain the association is insignificant. Again, a visual inspection indicates that these three countries are insignificantly different from each other and Great Britain is insignificantly different from Austria and Belgium.

### Additional covariates

For completeness, Table 3 reports descriptive statistics on all variables in the prevalence and inequality models. Average CES-D 8 was 4.92 and 15% of the sample lived in poor quality housing. Average age was 48 and 49% were male. 64% of the sample were either married or in a civil partnership. There were children at home in 37% of households. The mean and median number of years of full-time education was 13. The average equivalent household income was €26,969, ranging from €3,806 to €109,100. 14% of the sample report that living on present income is difficult or very difficult.

Regarding main activity, 53% of the sample were in paid employment, 8% were in education, 8% were either unemployed or disabled and 24% were retired.

Regarding early-life experience, 38% stated that severe financial difficulties in the family when growing up were at least sometimes present, and 40% stated that serious conflict in the household when growing up was at least sometimes present.

The mean number of close friends was 4.1, with standard deviation of 2.86, and 36% of respondents reported low levels of social activity. 21% reported that they or a household member had been the victim of a burglary or assault in the last five years and 83% felt that their area was safe or very safe to walk alone after dark. Trust in other people averaged 5.61 with a standard deviation of 2.19.

**Table 3:** Summary statistics

Variable	Mean	SD	Min	Max
CES-D 8	4.92	3.70	0	24
Housing Quality	0.15	0.35	0	1
***Demographic***				
Age	48.57	18.24	14	114
Age^2^	2691.83	1841.06	196	12,996
Male	0.49	0.50	0	1
Married or civil partnership	0.64	0.48	0	1
Number in household	2.64	1.37	1	13
Children at home	0.37	0.48	0	1
***Socio-Economic***				
Years of Education	13.29	3.89	0	50
Equivalent income	26,989	13,865	3,806	109,100
Financial Strain:				
Living comfortably on current income	0.41	0.49	0	1
Coping on current income	0.45	0.50	0	1
Finding it difficult/very difficult to cope on current income	0.14	0.35	0	1
Main Activity:				
Paid Employment	0.53	0.50	0	1
Education	0.08	0.27	0	1
Welfare (unemployed/disabled)	0.08	0.27	0	1
Retired	0.24	0.43	0	1
Other	0.07	0.26	0	1
***Early Life Experience***				
Growing up your household experienced:				
Severe financial difficulties	0.38	0.49	0	1
Serious conflict between people	0.40	0.49	0	1
***Community***				
Number of close friends	4.12	2.86	0	13
Below average participation in social activity compared to others	0.36	0.48	0	1
Victim of burglary or assault in last 5 years (anyone in household)	0.21	0.41	0	1
Local area feels safe to walk alone after dark	0.83	0.38	0	1
Trust in people	5.61	2.19	0	10

Source: ESS, 2015. Authors’ calculations

Table 4 reports on the covariates associated with the prevalence of depressive symptoms. All demographic variables are significant, except age. Males, being married, and number in the household are all negatively associated with depressive symptoms while presence of children in the household is positively associated with depressive symptoms. Typical measures of socio-economic status – years of full-time education and income - are insignificant but a third measure, financial strain, is positively correlated with depressive symptoms as levels of strain worsen. Regarding main activity, being in education, or being unemployed or disabled are positively associated with levels of depressive symptoms compared with being in paid work.

Those who report growing up in households that had severe financial difficulty at least some of the time or who experienced serious conflict among household members at least some of the time have a positive, if modest, association with depressive symptoms. Among the positively framed community and social capital variables – number of close friends, trust in people, perception of local area as safe for walking after dark – are negatively correlated with depressive symptoms and the negatively framed ones – below average level of social activity and a member of the household being the victim of an assault or burglary relatively recently – are positively associated with depressive symptoms.

The last two columns of Table 4 report inequalities in depressive symptoms. Among the demographic variables, age is associated with increased inequalities in depressive symptoms, but at a decreasing rate. Presence of children at home is also associated with increases in inequalities and number of household members has the opposite association. Gender and marital status are insignificant.

Again, years of education and income have no statistically significant association with the concentration index, but the third measure – financial strain – is significant. The direction of its association differs by category, with ‘coping’ associated with higher inequality relative to ‘living comfortably’ – contrary to expectations – and ‘finding it difficult’ associated with lower inequality in depressive symptoms – as expected. Amongst the respondents’ main activity only those who were unemployed or disabled had a statistically significant association. These were associated with an increase in inequality relative to paid employment.

The results indicate no association between the two early-life experience variables and the concentration index. Only three of the community variables, low level of social activity, trust in people and perception of area after dark are significant. The associations between all three and the concentration index are in line with expectations.

In order to provide context to the relationship between poor housing quality and the concentration index, we examine relative magnitudes of effect. The marginal association between poor housing quality and the concentration index is at its lowest for the Netherlands (-0.0542) and there are three covariates with more negative coefficients -age at the sample average age of 49 is (-0.07987 = 0.0031(49) + 0.00003(49^2^)), ‘difficult or very difficult to cope on present income’ is -0.0936 and being unemployed or disabled (welfare) of -0.0853.

**Table 4:** Additional factors associated with depressive symptoms and inequalities in depressive symptoms

Variable	Negative Binomial Regression		RIF-EI	
	Coef.	P > z	Coef.	P > z
Age	0.9990	0.749	-0.00310	0.013
Age^2^	1.0000	0.217	0.00003	0.019
Male	0.8784	0.000	-0.00134	0.899
Married or civil partnership	0.8285	0.000	0.01917	0.304
Number in household	0.9819	0.000	0.00695	0.009
Children at home	1.0291	0.092	-0.02163	0.041
Years of education	1.0010	0.704	0.00076	0.441
Equivalent income	1.0109	0.613	0.01500	0.586
Financial Strain (ref cat: living comfortably)				
Coping on present income	1.1503	0.000	0.05264	0.001
Finding it difficult or very difficult to cope on present income	1.3813	0.000	-0.09364	0.000
Main Activity (ref cat: paid employment)				
Education	1.0924	0.000	0.00997	0.557
Welfare (unemployed/disabled)	1.2659	0.000	-0.08531	0.012
Retired	0.9627	0.132	-0.00663	0.558
Other	1.0629	0.021	-0.01303	0.385
Growing up your household experienced:				
Severe financial problems	1.0780	0.000	-0.00594	0.373
Serious conflict	1.1684	0.000	-0.01640	0.110
Number of close friends	0.9848	0.000	0.00028	0.752
Below average participation in social activity compared to others	1.2537	0.000	-0.02950	0.069
Victim of burglary or assault in past 5 years	1.0756	0.000	-0.01250	0.247
Area feels safe to walk alone after dark	0.8749	0.000	0.03215	0.066
Trust in people	0.9816	0.000	0.00360	0.024
Constant	4.5864	0.000	-0.16958	0.518
n	15,479		15,479	
R^2^	0.043		0.04440	

Source: ESS, 2015. Authors’ estimates

Note: Housing Quality, Country-level fixed effects and the interaction of housing quality and country are suppressed. Negative binomial regression results are presented as incidence rate ratios. RIF-EI = Recentered Influence Function – Erreygers Index

## Discussion

While there is a growing body of empirical research on housing quality and physical health, there is somewhat less on mental health [66–69], and less again on mental health inequalities [70]. This study examined inequalities in depressive symptoms in nine Northern European countries that had similar median equivalised household incomes and similar levels of income inequality. Each one also had a relatively comprehensive welfare state. The study benefitted from the use of the same survey instrument across all countries, facilitating the direct comparison of observed levels of depressive symptoms and inequality in depressive symptoms. An analysis of the factors associated with prevalence and inequality in depressive symptoms focused on their association with housing quality. To provide context to the main findings, the study also examined demographic, socio-economic, early-life experience and community-related factors associated with depression.

First, the median prevalence of depressive symptoms was 4, but there was a higher prevalence among poor people in all countries. The pooled average Erreygers Index was − 0.066, and it varied from Austria with (-0.04) to France (-0.079). While comparable studies are limited, Costa-Font and Gil (2008) found a relative concentration index for doctor diagnosed depression in Spain that varied from − 0.1090 to -0.1698 depending on number of covariates included [15]. Xu et al. (2016) found a relative concentration index of -0.0645 in depression for the elderly in China [19].

Poor housing quality is associated with an increase in prevalence of depressive symptoms in all countries. For inequalities in depressive symptoms, poor housing quality was associated with higher levels of the EI in six of the nine countries, lower in two and no relationship in one. No pattern could be detected with respect to region, cultural similarities, comprehensiveness of mental health system or housing system. In six on the nine countries studied, poor housing quality had a greater negative association with inequality in depressive symptoms than all other covariates except for age, financial strain and being unemployed/disabled, at the 5% level. This finding indicates the relative importance of the association and suggests that further research on the relationship would be welcome.

This result adds to literature like Shah et al. (2018) on the effect of housing quality on mental health inequalities [68]. It also mirrors work on housing quality and physical health inequality. For instance, Nie et al. (2022) and Cai et al. (2017) studied the association between housing and physical health inequalities in China, using the same RIF-decomposition method as in this paper [8, 37]. These papers also found that poor housing quality increased health inequality. The housing quality in Europe is, however, of a higher general standard than that examined in Nie et al. (2022) - where access to tap water and electricity/gas and indoor flush toilets were among the indicators assessed – so the studies are not directly comparable [8]. In Spain, Urbanos-Garrido (2012) [9], using the method for concentration index decomposition proposed by Wagstaff et al. (2003) [33], and a set of community indicators similar to those used in this paper, as well as the usual socio-economic and demographic indicators, found that poor housing conditions had a considerable effect on physical health inequality.

This study contributes to the current debate in Europe surrounding inadequate housing, home construction and retrofitting policies [71] and further suggest that meaningful policies should be focused on low income households. Low income increases the risk of fuel poverty which can lead to cold, damp and mould within dwellings [72]. Fuel poverty is rising in many countries because of higher fuel prices that are not offset by energy efficient improvements in low-income households [73]. In the short-term, providing electricity or fuel subsidies can alleviate the burden of paying for energy for low-income groups especially during winter-time [74]. However, calls have been made for greater investment in structural interventions that make homes more energy efficient (e.g. Energy Efficiency Façade Retrofitting) or heating system improvements given that these can have greater long-term positive impacts [73, 75] and that mental health tends to improve following improvements in warmth and energy efficiency within the home [21, 26] and following fabric works [23].

### Limitations

Despite the contributions of this study, there are some potential limitations that bear mentioning. The ESS data is taken from 2015 as this is the only wave currently that contains information on housing problems and depression, and it provides a comparative Pan-European dataset which has harmonized design, sampling and data collection methods. Over the past decade, depression rates in the EU have increased slightly (from 6.9% to 2014 to 7.2% in 2020 [3] and housing problems such as poor energy efficiency/poor insulation of the dwelling continue to be experienced by about a quarter of the EU’s population [71]. Moreover, we cannot ignore the effect of the Covid-19 pandemic in exacerbating inequalities around the world more recently [76].

The ESS generated a usable sample of only 1,720 on average for each of the nine countries studied. Consequently, sample size limitations preclude the use of multiple interaction terms, hierarchical models or similar modelling approaches. Larger sample sizes would allow decompositions of all contributors to inequality at country level and is a fruitful avenue for future research.

Due to the cross-section design of the ESS, reverse causality cannot be ruled out. For example, people with depression may be less likely to seek social support from others or engage in social activities [77]. Further evidence suggests depression is linked to adverse outcomes in educational achievements and economic performances [78]. The study design does not allow causal interpretation of the findings. Rather what we present are associations between the explanatory variables and depression, which was our aim. Furthermore, housing situation may be a symptom of adverse or worsening living and working conditions leading to mental health deterioration over a period of time. This highlights the importance of designing and implementing longitudinal surveys that track changes in individual depression over time.

## Conclusion

The study used the same data capture methods to examine the degree of socio-economic inequalities in depressive symptoms in nine comparable European countries. Housing quality was a strong contributory risk factor to inequalities. Given the comparative dearth of evidence of the association and putative effect of poor housing quality on depression and inequality in depression, this study is a valuable contribution to the field.

### Electronic supplementary material

Below is the link to the electronic supplementary material.

Supplementary Material 1

## Data Availability

European Social Survey, Round 7, 2014. This is publicly available from the following link: European Social Survey | European Social Survey (ESS).
