# Supplementary material for: A happy home? Socio-economic inequalities in depressive symptoms and the role of housing quality in nine European countries
Source: BMC Public Health. 2023 Nov 8;23:2203. doi: 10.1186/s12889-023-17070-z (PMC10634013; doi:10.1186/s12889-023-17070-z)
Supplement: Supplementary file 1 — Supplementary Material 1 [file 12889_2023_17070_MOESM1_ESM.docx]

**Supplementary File 1**

**Justification of Choice of Country**

Figure S1 presents Median Income for ESS countries in 2015.

**Figure 1 Median Income for ESS countries in 2015**

Source: ESS, 2015.

Note: No income data were available for Estonia. Israel was excluded as it is not an unambiguously European country.

An assessment of median equivalent incomes across the ESS indicates a considerable range – from Switzerland at c€50,700 to Lithuania at c€4,200. Switzerland and Norway are outliers on the high-income side. There is a step change from Norway to Denmark from c€48,000 to c€31,500 (a 1:0.66 ratio). The next step change is arguably Ireland to Spain from c€17,800 to c€11,800 (again a 1:0.66 ratio). Below that while the absolute differences in median are small the proportionate ones are more significant. The group of countries with the most similar levels of median income appear to range from Denmark to Ireland on this criterion.

Figure S2 presents Gini coefficient data for ESS countries.

Gini coefficients for ESS countries

Source: ESS, 2015.

Note Estonia and Israel excluded.

An assessment of income inequality as measured by the Gini coefficient, indicates that its range is from 0.35 in Ireland to 0.199 in Czech Republic. There is a steady decline from Ireland to Switzerland and the slight decline from Switzerland through to the Czech Republic. In fact, the decline from Ireland to Switzerland (0.079) exceeds the decline from Switzerland to the Czech Republic (0.078). This would indicate that on this criterion all but Ireland, Great Britain, Portugal and Spain should be included in the analysis.

Examining both criteria, Spain and Portugal are clearly excluded falling below the income threshold and exceeding the income inequality one. The five other low income countries are excluded under the median income criterion. Although it is included under median income criterion, Ireland the lowest ranked country under this criterion and the highest ranked country under the Gini criterion so we chose to exclude it. Great Britain has a higher median income than Ireland’s and a lower income inequality coefficient so we chose to include it.

Therefore, the country choice turns out to be one where median income vary from approximately €20,000 to €30,000 - except Denmark which is just above threshold - and Gini varies between 0.20 and 0.30.
